# Construction of China’s climate civil public interest litigation system–from the perspective of public health protection and medical assistance

**DOI:** 10.3389/fpubh.2026.1860605

**Published:** 2026-05-18

**Authors:** Jiezhong Chang, Gang Zeng, Wei Hu

**Affiliations:** 1 The Center for Modern Chinese City Studies, East China Normal University, Shanghai, China; 2 School of Geographic Sciences, East China Normal University, Shanghai, China; 3 Department of Law, School of Economics and Management, China University of Geosciences (Beijing), and the Ministry of Education Laboratory of Philosophy and Social Sciences for Mineral Resource Security Governance, Beijing, China

**Keywords:** carbon justice, climate civil public interest litigation, ecological environment code, medical and health big data, precautionary principle, public health, right to public climate health

## Abstract

To safeguard public health, ecological and environmental rights, and ecological security, China enacted the *Ecological Environment Code* in 2026, which includes a dedicated chapter on climate change. However, climate civil public interest litigation addressing climate-related health damage still relies on general ecological and environmental litigation provisions and lacks a specialized institutional framework. Climate change not only disrupts ecosystems and carbon cycle balance, but also increases disease burden, intensifies pressure on medical resources, and exacerbates health inequalities, posing systemic challenges to public health and medical security systems. For example, extreme weather events raise the incidence of cardiovascular and respiratory diseases, ecological imbalance promotes the spread of vector-borne and infectious diseases, and vulnerable populations face increased health risks and medical burdens. Although closely related to ecological destruction and air pollution public interest litigation, climate civil public interest litigation differs in its theoretical foundation, value objectives, normative scope, and legal methods, thus requiring independent institutional design. In China, courts often apply general environmental liability rules, resulting in insufficient scientific precision and inadequate responses to climate-related health risks and medical cost burdens. This paper fills the research gap by proposing a specialized climate civil public interest litigation system within dedicated climate change legislation, grounded in the risk prevention principle and the right to public climate health. It constructs a climate–health–medical big data-supported evidence rule system, improves carbon emission monitoring rules, and designs a prevention-oriented civil liability framework supplemented by relief mechanisms, including medical insurance, while strengthening coordination with existing environmental public interest litigation systems and provides an interdisciplinary bridge between climate governance, public health protection, and multilevel medical security.

## 1 Introduction

As the threat of climate change to global ecosystems and human health intensifies, climate civil public interest litigation has become an important means of addressing the climate crisis in many countries. Chinese judicial organs have carried out judicial work on climate civil public interest litigation in accordance with the *Environmental Protection Law* amended in 2014 and the *Civil Procedure Law* amended in 2017. In order to protect the ecological environment, prevent and control pollution and other public hazards, safeguard public health and ecological and environmental rights and interests, maintain ecological security, and promote green and low-carbon development, the National People’s Congress adopted the *Ecological Environment Code* in March 2026 (1). To protect the public ecological environment through litigation, the Code establishes an ecological and environmental civil public interest litigation system. To protect public climate interests, the Code establishes a dedicated chapter on addressing climate change, but the legal provisions on climate civil public interest litigation are not yet specific. At present, China’s climate civil public interest litigation is mainly initiated by qualified social organizations and procuratorial organs against acts such as irregular greenhouse gas emissions and illegal destruction of carbon sinks that harm public climate interests. The legal rule system constructed by the state around climate civil public interest litigation constitutes the climate civil public interest litigation system.

Climate change’s destruction of ecosystems not only affects the service functions of forests, wetlands, oceans, and other ecosystems—such as carbon sinks, climate regulation, and biodiversity maintenance—that support human health; once these functions are damaged, they will directly amplify the threat of climate risks to health, affect public health (especially vulnerable populations), increase health maintenance and medical burdens, and also exert far-reaching impacts on the operation of medical systems by changing disease spectrum structures and medical service demands. Examples include heatstroke caused by heatwaves, water source pollution and malnutrition caused by ecological degradation, and increases in infectious diseases caused by the spread of disease-vector insects. Therefore, examining climate civil public interest litigation from the perspective of ecological security, public health, and medical assistance has more prominent practical significance.

Interdisciplinary public health literature provides an important empirical and methodological basis for this legal inquiry. The WHO has emphasized that climate change affects the social and environmental determinants of health, including clean air, safe drinking water, sufficient food, and secure shelter (2). The Lancet Countdown has continuously tracked the links between climate change and health through multi-indicator monitoring, including heat-related mortality, infectious disease transmission, food and water security, health-system resilience, and the economic costs of climate-related health impacts (3). The 2025 China report of the Lancet Countdown further shows that climate-related health risks in China are increasing and that evidence-based, city-specific climate-health actions are needed (4). In addition, the IPCC has emphasized that climate risks cut across sectors and regions and affect both natural and human systems, while recent attribution research has increasingly connected extreme events with socioeconomic and health impacts (5, 6). These studies indicate that climate-related health damage is not merely an ecological consequence, but also a public health, medical security, and health economics issue.

According to the authors’ search of the Sabin Center Climate Change Litigation Database, retrieved on October 8, 2025, 4,280 climate litigation records were identified globally, while only four China-related climate litigation cases were recorded (7). In contrast to the rapid growth of global climate litigation, most carbon-related cases in China remain attached to other environmental litigation systems, and specialized climate-health relief remains insufficient. In this context, constructing a specialized climate civil public interest litigation system has become the proper meaning of addressing climate issues. However, some studies take a cautious attitude toward climate civil public interest litigation systems based on tort, arguing that the tort path does not conform to China’s national conditions (8), that the “de-substantiation” feature weakens the practical value of the rights path (9), and that its functions cannot surpass the individualized, ex-post relief model of traditional tort law (10). Yet some scholars have argued from rights theory for the feasibility of new rights foundations in litigation (11). In terms of specific paths for institutional construction, more scholars have begun to explore the feasibility of incorporating it into the existing environmental public interest litigation system, advocating environmental civil public interest litigation from the perspectives of risk prevention, state ownership of natural resources, and the homology of air pollutants and greenhouse gasses (12–14). Chuanxuan Li, drawing on foreign experience, sought an institutional positioning for climate justice within the existing environmental judicial framework (15). However, research has not yet adopted a specialized institutional construction approach. Quanrong You argued that carbon-related cases differ from traditional environmental resource cases and that it is necessary to break path dependence on traditional judicial specialization through carbon-related judicial specialization (16). Nan Guo argued that carbon-related disputes should be integrated into the specific type of climate change response cases (17). This is a useful exploration of the specialization of carbon-related cases, but it has not yet fully integrated the rules of specific environmental litigation systems, resulting in a lack of concrete methodological guidance for institutional construction. However, existing studies have not yet constructed a specialized climate civil public interest litigation system from the perspectives of public health protection, medical assistance, and medical security. Domestic scholarship has mainly focused on the relationship between climate litigation and traditional environmental public interest litigation, the feasibility of tort-based climate liability, carbon market regulation, and judicial specialization of carbon-related cases (8–17). Internationally, while climate-health research, climate attribution science, and health economics have developed rapidly (2–6), few studies have explored how judicial mechanisms can integrate medical and health big data, medical insurance, and medical assistance into climate damage relief systems.

This paper therefore fills a specific research gap: existing studies have not constructed a specialized climate civil public interest litigation system from the combined perspectives of public climate health rights, medical assistance, and climate–health–medical data governance. The practical urgency is particularly clear in China. Although carbon-related cases are numerous and closely related to ecological protection, China still lacks a specialized adjudication framework for climate civil public interest litigation; adjudication remains inconsistent across case types, and health-oriented relief mechanisms, including medical insurance-based relief, remain underdeveloped. This paper responds to that gap by proposing a specialized institutional framework that connects climate governance, public health protection, medical assistance, and multilevel medical security.

## 2 Methods

This study employs a qualitative legal and policy research design combining doctrinal legal analysis, case-based examination, comparative reference, and institutional design. First, it interprets the *Ecological Environment Code* (2026), with particular attention to Articles 5, 6, 147, 1,028, 1,029, and 1,075, in order to identify the normative basis for integrating climate governance, public health protection, public interest litigation, and data-supported adjudication (1). Second, it examines representative carbon-related cases issued by the Supreme People’s Court and the Supreme People’s Procuratorate, together with China-related climate litigation records in the Sabin Center Climate Change Litigation Database, to identify current gaps in adjudication, evidence rules, and health-oriented relief (7). Third, it draws on comparative climate litigation experience and interdisciplinary literature from public health, health economics, and climate attribution science to develop an institutional design for climate civil public interest litigation in China (2–6).

The case analysis is not intended as a quantitative empirical study. Rather, it is used to identify doctrinal and institutional problems in existing judicial practice, including the lack of specialized litigation objects, insufficient climate–health causation rules, limited use of medical and health data, and the absence of medical insurance-based relief mechanisms. On this basis, the paper proposes a specialized climate civil public interest litigation framework oriented toward public climate health rights and multilevel medical security.

## 3 Independence of the climate civil public interest litigation system and its value objectives in protecting public health, providing medical assistance, and other public climate rights and interests

### 3.1 Association and differentiation between climate civil public interest litigation and ecological destruction and pollution prevention public interest litigation

Some acts that affect climate change are closely related to ecological destruction in fields such as forests, fisheries, minerals, land, and water resources (18). The mechanism is that excessive greenhouse gas emissions or acts that damage carbon sinks disrupt the carbon cycle balance and climate regulation service functions of the atmospheric ecosystem, causing systemic damage or threats to global ecosystems and human health. For example, forest reduction will intensify local climate extremes and increase respiratory diseases and heat-related deaths; wetland destruction may expand the transmission range of mosquito-borne diseases. Whether ecological destruction acts or greenhouse gas emission acts, both cause damage through natural ecosystems as the medium (19). In September 2013, Indonesia’s Ministry of Environment and Forestry filed a tort lawsuit against PT Merbau Pelelawan Lestari for illegal logging that damaged the forest’s carbon sink capacity; the Supreme Court ordered the defendant to compensate for the restoration costs of climate damage (20). Although the case was regarded as an incidental climate litigation case, it promoted Indonesia’s domestic climate governance process (21). This logical reasoning is worth referencing when China formulates dedicated climate change response legislation.

Some acts that affect climate change are closely related to air pollutant emission acts. In industrial activities, greenhouse gasses and air pollutants are both produced by fossil fuel combustion; once emissions exceed the environment’s self-purification capacity or climate resilience (22), they will produce ecological and environmental impacts and endanger human health. In developing countries, emitting carbon dioxide into the atmosphere does not necessarily reduce atmospheric environmental quality (23), but controlling air pollutant emissions often reduces fossil fuel consumption and has a positive correlation with greenhouse gas emission reduction, reflecting the synergistic effect of pollution reduction and carbon reduction (14). In the U. S. case *Massachusetts v. EPA*, although the U. S. federal court ruled that carbon dioxide is an air pollutant (24), the U. S. Environmental Protection Agency has always adopted a dual-track approach to greenhouse gasses and traditional air pollutants (25). In China, Article 5 of the 2026 *Ecological Environment Code* requires “coordinated promotion of carbon reduction, pollution reduction, green expansion, and growth,” treating greenhouse gasses as independent ecological and environmental impact substances and not including them in the scope of air pollutants; therefore, it is inappropriate to bring public interest litigation caused by greenhouse gas emissions into the scope of air pollution civil public interest litigation.

### 3.2 Legal construction and normative basis of the right to public climate health

The right to public climate health can be understood as a collective and preventive legal interest derived from the broader right to a clean, healthy, and sustainable environment. In 2022, the United Nations General Assembly recognized the right to a clean, healthy, and sustainable environment as a human right, providing an important international normative background for understanding climate-related public health interests (26). In the context of climate civil public interest litigation, the right to public climate health refers to the public’s interest in maintaining a stable climate system and ecosystem service functions that are necessary for protecting life, health, and access to essential medical assistance.

Unlike an individualized medical claim after a specific disease has occurred, the right to public climate health emphasizes the prevention of systemic and population-level health risks caused by climate change. Its beneficiaries include present generations, future generations, and vulnerable groups that are more exposed to heatwaves, infectious diseases, food and water insecurity, and limited access to medical resources (2–5). This right is collective because climate-related health risks usually affect an indeterminate number of people; preventive because climate-health damage is often delayed, cumulative, and difficult to reverse; and relational because it connects ecological interests, public health interests, and medical security interests.

The normative basis of this right can be constructed through a systematic interpretation of the *Ecological Environment Code*. Article 5 requires the state to coordinate industrial restructuring, pollution prevention and control, ecological protection, and climate change response, and to promote carbon reduction, pollution reduction, green expansion, and growth in a coordinated manner. Article 6 further establishes prevention as a basic principle of ecological and environmental protection. Article 147 provides the procedural basis for qualified social organizations to initiate ecological and environmental civil public interest litigation, while Article 1,075 authorizes procuratorates and qualified social organizations to bring litigation against acts that pollute the environment, destroy ecology, or otherwise damage social public interests (1). These provisions jointly support the conclusion that climate-related acts endangering public health may be brought within the scope of ecological and environmental civil public interest litigation, but they also reveal the need for more specialized climate litigation rules.

Accordingly, the object of climate civil public interest litigation should be clarified in three progressive layers: first, the interest in climate system stability, including carbon cycle balance, carbon sinks, and climate regulation functions; second, the public health interest, including the prevention of heat-related diseases, respiratory and cardiovascular diseases, vector-borne diseases, and nutrition or water-security-related health risks; and third, the medical security interest, including the reduction of avoidable medical burdens, protection of vulnerable populations’ access to medical assistance, and rational use of medical insurance funds. This layered structure helps transform abstract climate interests into legally reviewable public interests and connects climate litigation with public health governance and multilevel medical security.

A brief comparative review further shows that major climate litigation models have addressed public health or welfare concerns to different degrees, but few have directly integrated medical-cost allocation or medical insurance relief into climate litigation.

As shown in Table 1, existing climate litigation models have made important contributions to emission regulation, rights protection, adaptation-cost allocation, and ecological restoration. However, they rarely provide a direct institutional pathway for integrating public health damage, medical assistance, and medical insurance into climate litigation. This comparative gap further supports the necessity of constructing a specialized climate civil public interest litigation system in China.

**Table 1:** Comparative approaches to climate litigation, public health, and medical costs.

Jurisdiction/case	Main legal route	Treatment of public health or medical costs	Implication for China
United States/*Massachusetts v. EPA* (24)	Administrative and regulatory litigation under the Clean Air Act	Greenhouse gasses were treated as air pollutants for regulatory purposes; public health and welfare were addressed mainly through regulatory authority rather than medical-cost compensation.	This case is useful for linking greenhouse gas regulation with health protection, but it provides limited guidance for designing medical insurance-based climate relief.
Netherlands/*Urgenda Foundation v. State of the Netherlands* (48)	Rights-based climate litigation and state duty of care	Climate risk was framed through human rights protection, including life and well-being, but the remedy focused on emission reduction rather than individualized medical relief.	This case supports preventive climate litigation based on public rights, but it does not directly solve health-cost allocation.
*Germany/Luciano Lliuya v. RWE AG* (49)	Civil liability and climate adaptation-cost claim	The case illustrates an attempt to seek proportional contribution to climate adaptation costs; medical costs were not the central claim.	This case is useful for exploring causation and proportional liability, but China still needs specialized rules for public health and medical security interests.
Indonesia/*Ministry of Environment and Forestry v. PT Merbau Pelelawan Lestari* (20)	Environmental tort related to forest carbon sink destruction	Relief focused on restoration costs for ecological and climate-related damage rather than medical assistance.	This case shows how carbon sink damage may be linked with ecological restoration, but health-oriented relief remains underdeveloped.
China/Current carbon-related cases (7, 32)	Environmental public interest litigation and ecological damage compensation	Existing cases mainly rely on ecological restoration, compensation, carbon sink purchase, or technological transformation; health relief and medical insurance mechanisms remain insufficient.	This demonstrates the need to construct a specialized climate civil public interest litigation system integrating public health, medical assistance, and medical security.

### 3.3 Independence of the climate civil public interest litigation system and the prominent value of protecting public health and other public climate rights and interests

From the theoretical foundation perspective, the climate civil public interest litigation system is built on a specialized theory. Given that the relationship between acts causing climate change and the resulting ecological and environmental damage and human health damage has characteristics of asynchrony, complexity, lag, and concealment, the climate civil public interest litigation system takes public climate rights and interests and the risk prevention principle as its theoretical support, shifting the focus from “determined damage” to “possible risks” (27). Public climate rights and interests refer to the right of contemporary and future citizens to enjoy a suitable environment and climate (28). Their connotation has expanded from traditional specific environmental elements such as air, water, and soil to the overall ecological environment with the climate system at its core. Its ultimate foothold is to protect the public’s health rights and interests under the support of a sound ecosystem, as well as to ensure that populations suffering health damage receive timely and effective medical assistance. This is not entirely consistent with the theoretical foundation of the traditional ecological and environmental civil public interest litigation system.

From the value objectives perspective, the climate civil public interest litigation system aims to protect public health and other public climate rights and interests, promote medical assistance for populations suffering health damage, achieve corrective justice and distributive justice from the domestic to the international level and from the intra-generational to the inter-generational level (29), and realize climate governance goals with potential efficiency gains by preventing systemic risks before they generate large-scale ecological and health losses, reflecting human rights value, justice value, and efficiency value that transcend traditional environmental litigation (see Table 2).

**Table 2:** Value objectives: traditional ecological and environmental civil public interest litigation vs. climate civil public interest litigation.

Dimension	Traditional ecological and environmental civil public interest litigation system	Climate civil public interest litigation system
Human rights value	Emphasizes contemporary persons’ environmental rights, focusing on the basic right of citizens in specific regions to enjoy a healthy living environment	Emphasizes the collective human rights of all humankind, guaranteeing everyone’s basic human right to survive and develop in a safe and good climate environment
Justice value	Mainly concerns distributive justice of environmental interests among contemporary persons, focusing on repairing already occurred ecological damage	Guarantees the interests of multiple eras (e.g., future generations), multiple regions (e.g., small island states), and multiple groups (e.g., vulnerable groups), reflecting the concept of fairness across time and space
Efficiency value	Restores specific ecological environments through individual case relief, focusing on damage compensation and local ecological restoration	Prevents systemic climate risks through litigation, promotes energy transition and policy change, and realizes low-carbon transformation of economic and social development
Ecological and health value	Focuses on ecological restoration in specific regions to protect local health rights and interests	Protects ecosystem service functions through litigation, prevents systemic climate-health risks, provides medical assistance to populations suffering health damage, and realizes protection of the health rights and interests of all humankind

From the legal adjustment scope perspective, although climate change can be included in the categories of environmental pollution or ecological destruction through expansive interpretation, environmental pollution and ecological destruction cannot encompass all carbon-related cases. In Chinese practice, carbon-related cases are not only diverse in type and overlap with one another, but some acts have not yet been fully identified and regulated. They include but are not limited to greenhouse gas emissions, construction of high-carbon projects, destruction of natural resource carbon sequestration functions, and acts that damage ecological and environmental public interests in the construction of zero-carbon parks, green buildings, and the national carbon market (30). This is inconsistent with the normative matters of traditional ecological and environmental law.

From the legal adjustment method perspective, in order to prevent the irreversibility of climate change, legislation and judicial intervention are often based on the “risk prevention principle” for acts that may cause climate damage, human health damage, or ecosystem damage. In Chinese judicial practice, carbon governance methods differ significantly from traditional environmental governance methods. For example, carbon market trading and pollutant discharge rights trading differ in object impact scope, governance objectives, and market scope; the carbon market is a key policy tool for China to achieve its “dual carbon” goals (i.e., carbon peaking and carbon neutrality) (31). In addition, China has formed unique adjustment methods such as product carbon footprints and enterprise carbon management, which are important standards for the identification and accountability of carbon-related acts and are inconsistent with traditional ecological and environmental law control methods.

From the perspective of public health governance, the value of the climate civil public interest litigation system is not only reflected in ecological and environmental protection but also in the timely and effective assistance of populations suffering health damage, reducing the disease burden caused by climate change to the state and society, alleviating payment pressure on medical security systems, and enhancing the resilience of public health systems to climate shocks. Therefore, this system should also be regarded as an important institutional tool connecting climate governance and public health governance.

### 3.4 The necessity for China to establish a specialized climate civil public interest litigation system

From the theoretical perspective, climate civil public interest litigation differs from traditional ecological and environmental civil public interest litigation and possesses independent legal attributes. In order to effectively promote climate civil public interest litigation, it is necessary to take the purpose of the *Ecological Environment Code* of safeguarding public health and ecological and environmental rights and interests as the starting point and, based on the provisions of ecological and environmental civil public interest litigation, specially establish a climate civil public interest litigation system for the protection of public health, medical assistance, and other public climate rights and interests when formulating dedicated climate change response legislation.

From the ecological and health perspective, establishing a specialized climate civil public interest litigation system is particularly necessary. It can repair damaged ecosystem service functions (such as carbon sinks, climate regulation, and biodiversity maintenance) through judicial means, effectively block the transmission path of climate change to public health risks, and achieve dual protection of ecological protection and public health rights and interests.

From the practical perspective, after the *Paris Agreement* was adopted, Chinese courts have concluded 1.12 million first-instance carbon-related cases, including cases involving economic and social green transformation, industrial structure adjustment, energy structure adjustment, carbon market trading, and other carbon-related cases (32). At present, carbon-related cases are numerous, growing rapidly, and highly correlated with other cases. However, carbon-related cases and dual-carbon cases are not normative case types (17). Other associated litigation systems lack a clear institutional foundation for achieving the “dual carbon” goals, making it difficult to form advantages in similar cases and unified adjudication rules, which is not conducive to specialized institutional construction. The *Environmental Resources Case Types and Statistical Norms (Trial)* created the new cause of action “climate change response cases” to uniformly manage “carbon-related cases.” In this context, constructing a climate civil public interest litigation system can achieve effective regulation of various carbon-related acts.

## 4 Limitations of China’s current rules on climate civil public interest litigation: deficiencies in the protection of public health, medical assistance, and other public climate rights and interests

In the “Green Development” Part IV of the 2026 *Ecological Environment Code*, a dedicated section on “addressing climate change” is established, indicating that “climate change” falls within “ecological environment.” Article 147 of the Code provides that qualified social organizations may file ecological and environmental civil public interest litigation, and this litigation should include climate civil public interest litigation; Article 1,075 states that where acts violate legal provisions by polluting the environment or destroying the ecology and thereby damage social public interests, people’s procuratorates or qualified social organizations may file lawsuits with people’s courts in accordance with law. Logically, people’s procuratorates and qualified social organizations may file ecological and environmental civil public interest litigation that includes climate change. These two provisions do not establish specialized legal rules for addressing climate change and therefore do not amount to establishing a specialized civil public interest litigation system for addressing climate change. In order to establish this specialized system, it is necessary to comprehensively analyze the problems existing in the current litigation rule system based on real judicial cases. To this end, this paper selects 10 carbon-related cases related to the climate civil public interest litigation system from the 21 typical cases issued by the Supreme People’s Court and the Supreme People’s Procuratorate before 2026, as well as the 4 cases counted in the Sabin Center for Climate Change Law database (7), to analyze the construction and application of China’s climate civil public interest litigation rules.

Before the promulgation of the 2026 *Ecological Environment Code*, the 2014 *Environmental Protection Law* did not explicitly provide for climate change. Therefore, in the above 10 cases, courts’ adjudication of climate civil public interest litigation cases was mainly attached to the traditional ecological and environmental damage civil public interest litigation system and often applied the relevant provisions of ecological protection and pollution prevention in the *Environmental Protection Law* according to case relevance (see Table 3). The scientificity and targeting of legal application were insufficient. Courts’ adjudication of public interest litigation cases addressing climate change is mostly concentrated in specific types such as forest resource cases and has not yet formed a specialized case-type system, resulting in failure to focus on core climate issues during legal application and difficulty in responding to the systemic and cross-domain requirements unique to addressing climate change. Nevertheless, the adjudication of such cases has played a positive role in achieving the “dual carbon” goals and promoting the state to fulfill its carbon emission reduction commitments (33).

**Table 3:** Specific characteristics of 10 typical carbon-related cases.

Case type	Legal basis	Liability forms	Fields involved
Air pollution civil public interest litigation; Ecological destruction civil public interest litigation; Ecological environment damage compensation litigation	Civil Code, Civil Procedure Law, Environmental Protection Law, Air Pollution Prevention and Control Law, Renewable Energy Law, Forest Law, Interim Regulations on the Administration of Carbon Emission Trading, Reform Plan for the Ecological Environment Damage Compensation System, Supreme People’s Court Interpretation on Several Issues Concerning the Application of Law in the Trial of Forest Resource Civil Disputes, Supreme People’s Court Interpretation on Several Issues Concerning the Application of Law in the Trial of Environmental Civil Public Interest Litigation Cases	Ecological restoration costs (payment of ecological and environmental damage compensation, appraisal and assessment costs) Alternative restoration (replanting and greening, off-site replanting, proliferation and release, eelgrass planting, labor compensation) Innovative liability (purchase of carbon sinks, implementation of technological transformation) Comprehensive measures (punitive damages, public apology)	Tampering with environmental monitoring equipment data, illegal fishing of aquatic products, illegal occupation of forest land, endangering nationally protected plants, illegal logging, theft of forest trees

This table only counts the content of environmental civil public interest litigation cases and the civil content in criminal incidental civil public interest litigation cases.

Judicial adjudication must be premised on clear legal provisions. The 2026 *Ecological Environment Code* provides basic provisions on addressing climate change and the ecological and environmental civil public interest litigation system, but it does not directly provide for climate civil public interest litigation regarding the protection of public climate rights and interests, including public health and medical assistance. Because more specific dedicated climate change response legislation has not yet been promulgated, the principled provisions on addressing climate change in the *Ecological Environment Code* are difficult to fully and effectively implement (34), which will cause climate civil public interest litigation to face four problems. These problems are reflected not only in the absence of a dedicated statutory cause of action, but also in inconsistent adjudicatory reasoning, insufficient recognition of climate-related health damage, and the lack of relief mechanisms capable of responding to medical burdens caused by climate risks. First, public climate interests and the public health interests they carry have not yet been established as independent legal interests, and health relief systems including medical insurance have not been provided for, resulting in climate-change-induced health risks being unable to enter the judicial review framework and climate damage being unable to become an independent type of legal infringement; this will affect the establishment of specialized rules for climate change public interest litigation. For example, in the so-called “first dual-carbon public interest litigation case” (35), the court adopted an evasive attitude toward the determination and adjudication of “climate public interest” damage, which will prevent health risks caused by ecological damage from receiving timely prevention. Second, associating climate change public interest litigation with ecological and environmental litigation tends to damage the scientificity and rigor of legal liability. Take the civil public interest litigation case of illegal fishing of aquatic products by Zhang and 14 others in Rongcheng City, Shandong Province as an example: the defendants drove two “three-no” fishing boats (i.e., vessels operating without registration certificates, without fishing permits, and without safety inspection certificates) to the Yellow Sea during the fishing moratorium and caught about 20,700 jin (approximately 10,350 kg) of Liang fish and Dao fish; the court ultimately ordered the planting of eelgrass. Although eelgrass has the function of maintaining the fish growth environment and objectively possesses certain carbon sequestration and oxygen release effects, the case is essentially not a climate case and failed to reflect the indirect protective effect of ecological restoration on public health. Third, in terms of the form of civil liability, courts, in addition to ordering compensation for losses, often apply “command-and-control” regulatory requirements such as ordering enterprises to limit production or suspend production when adjudicating, resulting in insufficient flexibility; for illegal acts such as exceeding intensity and total greenhouse gas emissions, there is a lack of necessary corrective civil liability and climate damage compensation provisions. In addition, ecological restoration, damage compensation, and other liabilities have not been linked to health risk assessment and medical assistance. Fourth, differentiated risk prevention, ecological and environmental restoration, public health protection, and other measures have not been provided for cases of carbon sink destruction and illegal greenhouse gas emissions, resulting in indirect damage to public health after the loss of ecosystem service functions being unable to receive timely relief (36). In addition, the current system has not yet established an integration mechanism and evidence rules for climate–health–medical data, limiting the application of medical health big data in climate civil public interest litigation.

## 5 Path for constructing a civil public interest litigation system oriented toward the protection of public health, medical assistance, and other public climate rights and interests

China is studying and formulating dedicated climate change response legislation on the basis of the *Ecological Environment Code*; this presents an opportunity to establish a specialized climate civil public interest litigation system in China. When constructing the specialized climate civil public interest litigation system, the system should be based on the basic provisions of ecological and environmental civil public interest litigation in the *Ecological Environment Code*. In dedicated climate change response legislation, a climate civil public interest litigation system should be established with the goal of protecting ecological security, public health, health assistance, and other public climate rights and interests; a climate–health–medical big data system and climate damage rule system should be established; climate damage relief mechanisms including medical insurance should be established; and the assisting functions of traditional ecological and environmental public interest litigation systems should be fully brought into play (30).

### 5.1 Constructing the climate civil public interest litigation system on the basis of the ecological and environmental civil public interest litigation system

#### 5.1.1 Clearly stipulating the risk prevention principle and public health, medical assistance, and other public climate rights and interests as the institutional Foundation of Climate Civil Public Interest Litigation

When interpreting the “environmental public interest” protected by the ecological and environmental public interest litigation system stipulated in the *Ecological Environment Code* (37), climate interests can be included in its regulatory scope. Therefore, the ecological and environmental civil public interest litigation system stipulated in the *Ecological Environment Code* serves as the normative foundation for constructing the specialized climate civil public interest litigation system.

The risk prevention principle should be interpreted together with the general and procedural provisions of the *Ecological Environment Code*. Article 5 provides the policy foundation by requiring coordinated governance of industrial restructuring, pollution prevention and control, ecological protection, and climate change response. Article 6 further confirms prevention as a basic principle of ecological and environmental protection. Article 147 provides the organizational basis for qualified social organizations to initiate ecological and environmental civil public interest litigation, while Article 1,075 provides the general litigation basis for procuratorates and qualified social organizations to bring claims where environmental pollution, ecological destruction, or similar acts damage social public interests (1). When climate-related acts create serious risks to public health and medical security, these provisions should be interpreted through the risk prevention principle to support earlier judicial intervention, rather than waiting for individualized and fully materialized health damage.

When constructing the specialized climate civil public interest litigation system, dedicated climate change response legislation should clearly stipulate the risk prevention principle and public climate rights and interests as the institutional foundation of climate civil public interest litigation. As a specialized system derived from the ecological and environmental civil public interest litigation system, the climate civil public interest litigation system possesses both the general characteristics of ecological and environmental civil public interest litigation and some of its own characteristics. The risk prevention principle emphasizes risk-preventive protection of special public climate interests such as public health, ecosystem services, ecological security, and food security. Public climate rights and interests emphasize that the law protects the public’s right to enjoy a suitable climate. Climate-harm acts not only infringe upon individuals’ basic rights to health and property but also infringe upon societal public health and other rights and interests. For such harmful acts, dedicated climate change response legislation must establish a specialized climate civil public interest litigation system when formulated, stipulate corresponding damage compensation and medical assistance liabilities, and correct climate illegal acts through judicial adjudication.

#### 5.1.2 Establishing a specialized climate civil public interest litigation system

Establish a specialized judicial system related to climate change. When formulating dedicated climate change response legislation, it is recommended that climate fairness, public health protection, and other related content be coordinated and set in the legislative purpose, basic principles, and policy framework (38), for example, by clearly stipulating the “risk prevention principle” and “public climate rights and interests” in the general provisions, and providing clauses such as “addressing climate change shall comply with the risk prevention principle” and “the people enjoy the right to live in a suitable climate,” thereby elevating climate rights and interests from “reflexive interests” or “derivative damage” to core rights and interests directly protected by law. On this basis, a climate change judicial system—including the climate civil public interest litigation system, climate change administrative public interest litigation system, and climate change criminal public prosecution system—should be established for climate rights and interests damage.

Improve specialized norms related to climate civil public interest litigation. First, incorporate climate civil public interest litigation into the ecological and environmental civil public interest litigation system. It is recommended that, in China’s *Provisions on Causes of Action in Civil Cases*, a fourth subcategory “climate civil public interest litigation” be added under the third-level cause of action “ecological and environmental protection civil public interest litigation,” and that when amending the *Supreme People’s Court Interpretation on Several Issues Concerning the Application of Law in the Trial of Environmental Civil Public Interest Litigation Cases*, “acts that damage the stability of the climate system and endanger public health” be included in the scope of damage to social public interests, thereby providing a legal basis for specialized institutional construction. Second, establish specialized prosecution clauses in the *Climate Change Response Law*, authorizing qualified organs and social organizations to file civil public interest litigation against illegal acts and pursue corresponding legal liabilities. Third, provide for carbon-related legal liabilities in relevant laws and regulations. Liabilities for illegal greenhouse gas emissions and illegal carbon sink damage should be strengthened in the *Ecological Environment Code*, *Forest Law*, *Interim Regulations on the Administration of Carbon Emission Trading*, and other laws and regulations related to environmental pollution, ecological destruction, and carbon markets, as well as in relevant judicial interpretations. Fourth, promulgate nationwide unified carbon measurement norms, establish a climate–health–medical linkage monitoring and evidence rule system, uniformly regulate the specific uses of compensation funds in carbon-related cases, and lay a technical foundation for the smooth operation of the climate civil public interest litigation system.

Continuously strengthen capacity building for climate civil public interest litigation and medical assistance. In order to make institutional operation smoother, it is recommended that, in accordance with the public interest litigation requirements of Articles 32 and 33 of the *Ecological Environment Code*, dedicated climate change response legislation establish a coordinated working mechanism for climate change litigation between administrative regulatory organs and judicial organs, and support climate and health monitoring institutions, medical insurance institutions, and medical assistance institutions in capacity building through funding support and professional training, thereby improving their ability to participate in and support climate civil public interest litigation and medical assistance.

#### 5.1.3 Constructing a climate–health–medical big data-supported evidence rule system

With the deepening of digital governance, data in the fields of climate change, public health, and medical security have been accumulated on a large scale, providing new evidence foundations and governance tools for climate civil public interest litigation. In this context, a linkage mechanism for climate change data, public health data, and medical security data can be constructed to promote the formation of cross-departmental data sharing and coordinated governance systems, breaking through the institutional bottlenecks of difficulty in proving causation and insufficient quantification of environmental damage in traditional litigation. Specifically, by integrating meteorological monitoring data (such as temperature, precipitation, and extreme weather events), environmental monitoring data (such as air quality and carbon emission data), disease monitoring data (such as incidence and mortality rates), medical service data (such as outpatient and emergency visits and hospitalization rates), and medical insurance payment data (such as expenditure structure and disease burden distribution), a multi-source data fusion climate–health risk analysis framework can be constructed. On this basis, dedicated climate change response legislation can stipulate the following legal mechanisms for climate civil public interest litigation:

Firstly, in terms of causation determination, scientific association between climate change factors and specific health damage outcomes can be achieved through time-series analysis, spatial correlation analysis, and attribution models, reducing the institutional obstacle of “difficulty in proving” in traditional tort law and improving the scientificity and explainability of judicial adjudication (5, 6).

Secondly, in terms of damage assessment, medical data and medical insurance data can be relied upon to conduct quantitative analysis of health damage caused by climate change, including increases in disease burden, rises in medical expenses, and labor losses, thereby providing objective bases for determining the scope and standards of damage compensation and promoting the transformation of climate damage liability from “abstract determination” to “fine-grained quantification” (39, 40).

Thirdly, in terms of public health governance, real-time data monitoring and risk early-warning models can link the climate civil public interest litigation system with public health emergency mechanisms, achieving a closed-loop operation of risk identification, judicial intervention, and policy feedback before and after extreme climate events, thereby enhancing the precision and foresight of public health systems in responding to climate shocks.

Fourthly, in terms of medical resource allocation and medical security decision-making, differentiated analysis of climate health risks across different regions and populations can provide data support for optimizing medical resource layout, health interventions for key populations, and adjustments to medical insurance payment policies, thereby promoting the formation of a “medical-prevention-insurance” coordinated governance system.

In addition, to ensure the standardized use of the climate–health–medical big data system in litigation, corresponding evidence rule systems should also be established, including legality review of data sources, transparency requirements for data processing, and the introduction of expert assistant systems, thereby guaranteeing the admissibility and probative force of data evidence. By constructing a data-driven evidence rule system, the scientificity and institutional effectiveness of climate civil public interest litigation can be significantly improved, and climate governance and public health governance can be promoted to achieve deep integration within the rule-of-law framework.

#### 5.1.4 Constructing a civil liability system featuring prevention as the mainstay and relief as the supplement, including medical insurance and medical assistance

The important position of preventive liability should be highlighted. According to the risk characteristics of climate change, prevention liability in climate civil public interest litigation can be divided into positive and negative types. The former requires carbon-related entities to take specific measures to prevent climate hazards, such as increasing dam heights in climate adaptation to eliminate threats of flooding to public health and life (10), and in climate mitigation taking measures such as industrial adjustment and upgrading, purchasing advanced equipment to reduce carbon emissions, and purchasing carbon sinks. These prevention measures should particularly focus on protecting ecosystem service functions and linking them with public health improvement effects—for example, restoring forest carbon sinks to reduce respiratory disease risks and restoring wetlands to reduce infectious disease transmission risks. The latter requires carbon-emitting enterprises to stop or partially stop carbon emission acts, including directly eliminating some industries or stipulating their production cycles through carbon emission impact assessments.

Relief liability can serve as an important supplement. At present, legislation on climate damage liability and compensation litigation in China is still in its early stage (41). If punitive damages are involved, excessive accountability should be avoided (42). In the criminal incidental civil public interest litigation case of illegal logging of forest trees by Jiang and Sheng in Suzhou City, Jiangsu Province, the procuratorial organ lawfully applied punitive damages and reasonably determined the compensation coefficient based on comprehensive factors such as the degree of forest ecological damage, the infringer’s subjective fault, and the restoration method. It can be seen that reasonable and lawful damage compensation, restoration of the original state, and punitive damages can relieve climate damage and strengthen the deterrent effect on carbon-related illegal acts. In order to bring into play the relief function of the climate change public interest litigation system for health and property damage, the following is recommended: firstly, define “damage to climate system stability and its major risks caused by excessive greenhouse gas emissions” as climate damage; secondly, design moderate damage compensation scope and standards for climate damage to prevent excessively broad scope and excessively high standards from affecting economic growth, and establish alternative restoration legal liability for climate hazards that are difficult to restore. In relief liability, ecological restoration liability should be particularly emphasized as being linked with health risk assessment—for example, while restoring forest carbon sinks, synchronous assessment of impacts on the respiratory health and nutritional health of surrounding residents should be conducted to ensure that ecological restoration produces obvious public health benefits; thirdly, establish climate-related medical insurance and medical assistance mechanisms, especially for vulnerable populations. The theoretical basis for integrating medical insurance into climate damage relief lies in the externalization of climate-related health costs. When climate-related acts increase disease incidence, emergency visits, hospitalization, or long-term treatment needs, part of the resulting cost is borne not only by individual patients but also by public medical insurance funds and public health budgets. Existing health economics research has shown that climate change is closely connected with sustainable healthcare and that rising temperature and CO_2_ emissions may increase government healthcare expenditure (39, 40). Therefore, medical insurance data can serve two functions in climate civil public interest litigation: first, as actuarial and evidentiary material for quantifying climate-related health losses; and second, as a basis for designing cost recovery or compensation mechanisms where legally attributable climate harm has increased public medical expenditure. This does not mean that medical insurance should replace the liability of climate-harm actors; rather, it means that medical insurance and medical assistance mechanisms can help identify, quantify, and allocate climate-related health costs within a prevention-oriented liability framework; fourthly, establish a consultation mechanism for climate damage compensation public interest litigation. Consultation may be presided over by the court, with participation by ecological and environmental departments, defendants, and others. In the consultation process, medical and health departments and health risk assessment experts may be introduced to jointly formulate restoration plans that balance ecological restoration and public health protection. The consultation system can resolve disputes promptly and avoid excessively lengthy litigation procedures.

In addition, the climate civil public interest litigation system should also be embedded in the overall framework of the multilevel medical security system: strengthening disease prevention functions at the basic medical level, enhancing climate risk response capacity at the public health level, and optimizing cost control and resource allocation at the medical security level, thereby reducing the systemic impact of climate change on medical system operation through institutional synergy.

### 5.2 Bringing into play the synergistic functions of traditional ecological and environmental civil public interest litigation Systems in Protecting Public Health, medical assistance, and other climate rights and interests

A notable characteristic of climate litigation cases in developing countries is the embedding of requirements for climate change mitigation and adaptation in fields such as environmental protection, land use, and natural resource protection, thereby promoting action by relevant fields and departments (43). Therefore, the climate civil public interest litigation system takes “climate core cases” as its regulatory focus. When climate change public interest litigation is unable to address many climate-related cases or lacks institutional capacity, related traditional ecological and environmental civil public interest litigation systems can play important synergistic roles in public health, medical assistance, and other climate interests.

Synergistic linkage with the ecological destruction civil public interest litigation system. When climate issues are not the core of a case but constitute a secondary or incidental dispute, it is an effective strategy to achieve prevention and relief of climate damage by relying on the ecological destruction civil public interest litigation system. When addressing ecological destruction acts such as illegal logging, illegal mining, and destruction of water bodies and wetlands, the above acts may be determined to have caused damage to the climate regulation functions of ecosystems and to public health. Through assessment of the carbon dioxide emission reduction volume within ecosystem function losses caused by carbon-related acts, compensation may be ordered for losses caused by the loss of ecosystem service functions and public health damage (44). This method can coordinate the relationship between climate interests and traditional ecological and environmental public interests (45), and construct a synergistic carbon-related liability accountability model (see Table 4).

**Table 4:** Carbon-related liability accountability model of the ecological destruction civil public interest litigation system.

Destruction field	Carbon-related accountability approach	Exploratory carbon-related liability forms	Ecological-health synergistic restoration or improvement
Destruction of fishery resources (e.g., harvesting aquatic plants)	Restoration and enhancement of blue carbon sinks	Planting eelgrass beds, establishing shellfish reefs, ordering purchase of forestry carbon sinks or direct compensation for carbon sink losses	Reduce water eutrophication and lower water-borne disease risks
Destruction of forest resources (e.g., felling trees)	Restoration and enhancement of green carbon sinks	On-site or off-site forest greening; ordering purchase of marine carbon sinks or direct compensation for carbon sink losses	Improve air quality and reduce incidence of respiratory diseases
Destruction of land resources (e.g., illegal occupation, pollution of cultivated land, grassland degradation)	Restoration of soil organic carbon pools	Remediation of polluted soil (restoration of carbon sequestration capacity); improvement of soil organic matter (increase in soil organic carbon storage)	Ensure food security and reduce nutrition-related health problems
Destruction of wetlands	Restoration of carbon sink functions of water bodies and wetlands	Restoration and reconstruction of wetlands, restoration of river and lake riparian vegetation, fixation of carbon in water bodies	Control disease vectors and reduce infectious disease transmission risks

Synergistic support with the environmental pollution civil public interest litigation system. According to the concept of synergistic efficiency of pollution reduction and carbon reduction, the climate civil public interest litigation system can fully draw on the mature value concepts, technical methods, and adjudication rules of the air pollution civil public interest litigation system. At the value concept level, for the public, air pollution—as a perceptible local environmental issue—is more likely to arouse resonance and social attention to public health protection. By building public awareness of the integrated coexistence of “blue sky” and “low carbon,” social consensus can be gathered for judicial protection of public health and other climate rights and interests. For carbon-related enterprises, facing dual judicial pressure of pollution reduction and carbon reduction, their illegal costs and legal risks will significantly increase, prompting them to turn to green technology investment and low-carbon transformation. For prosecution subjects, the professional capabilities in evidence collection and scientific determination accumulated in air pollution civil public interest litigation can be transformed to promote the improvement of the climate change public interest litigation system. At the scientific and technological level, air pollutants and greenhouse gasses have a high degree of symbiosis in emission sources and governance paths, providing a synergistic work foundation for green and low-carbon development. Fixed-source emission monitoring equipment can simultaneously collect greenhouse gas and conventional pollutant data, realizing unified monitoring, evidence collection, and systematic analysis of climate-environment data and providing scientific bases for post-treatment environmental synergistic governance. Atmospheric diffusion models and traceability technologies can also be used to provide methodological reference for determining the climate impacts of greenhouse gas emissions. Current research has quantified and traced the impact of specific heatwave events back to the historical emissions of 180 fossil fuel enterprises through systematic attribution methods (46), and broader attribution literature also shows that extreme event impact attribution is increasingly combining climate attribution with socioeconomic impact data (6). This indicates that the two types of civil public interest litigation for pollution reduction and carbon reduction can mutually support each other in scientific and technological evidence. At the judicial practice level, courts can draw on the judicial practice experience of the air pollution civil public interest litigation system to explore centralized jurisdiction and specialized adjudication for carbon-related cases (47), forming a more scientific climate governance model through institutional similarity. Through the synergy of pollution reduction and carbon reduction, scientific bases are provided for ecological-health governance. The above synergistic mechanisms not only help improve ecological governance effects but also reduce pressure on medical system operation by improving environmental health conditions and reduce medical payment pressure on parties and the government through medical assistance including medical insurance, thereby achieving synergistic optimization of climate governance and public health governance.

## 6 Conclusion

In the process of moving toward carbon neutrality, climate civil public interest litigation is an important legal defense line for protecting the public from health threats caused by climate change. Facing damage to public health, ecological security, and other public climate rights and interests, it is difficult to effectively punish climate illegal acts in accordance with traditional public interest litigation theory and the provisions on addressing climate change in the 2026 *Ecological Environment Code*. Therefore, a specialized climate civil public interest litigation system must be constructed. In terms of the construction path, specialized construction methods must be adopted on the basis of the ecological and environmental damage civil public interest litigation system, and the assisting role of traditional ecological and environmental civil public interest litigation must be brought into play synergistically. When formulating dedicated climate change response legislation on the basis of the *Ecological Environment Code*, it may be clearly stipulated that addressing climate change is included in the scope of acceptance of ecological and environmental civil public interest litigation, thereby providing direct legal basis for the filing and adjudication of climate change public interest litigation. In climate judicial practice, judicial organs should be encouraged to promote climate justice from conceptual consensus to adjudication consensus by issuing typical cases and special judicial interpretations, guiding the adjudication of relevant cases and promoting the effective implementation of the climate civil public interest litigation system. In addition, China should, in accordance with the provisions on international cooperation in addressing climate change in the *Ecological Environment Code*, strengthen international cooperation in climate justice with countries around the world, continuously protect public health and other public climate rights and interests, enable this system to promote the realization of China’s 2060 carbon neutrality goal, provide institutional support for the improvement of public health governance capacity and the construction of multilevel medical security systems, and contribute Chinese experience to global ecological civilization construction and public health security. The main contribution of this study lies in transforming climate civil public interest litigation from a primarily ecological remedy into an interdisciplinary governance mechanism that integrates public climate health rights, medical assistance, medical insurance, and climate–health–medical big data.

## Data Availability

The original contributions presented in the study are included in the article/supplementary material, further inquiries can be directed to the corresponding author/s.
